# Climate Projections and Pacific Lamprey Conservation: Evidence That Larvae in Natural Conditions May Be Resilient to Climate Warming

**DOI:** 10.3390/biology14010074

**Published:** 2025-01-15

**Authors:** Timothy A. Whitesel, Paul M. Sankovich

**Affiliations:** 1 Columbia River Fish and Wildlife Conservation Office, U.S. Fish and Wildlife Service, 1211 SE Cardinal Court, Vancouver, WA 98683, USA; 2 Columbia River Fish and Wildlife Conservation Office, U.S. Fish and Wildlife Service, 3502 Highway 30, La Grande, OR 97850, USA; paul_sankovich@fws.gov

**Keywords:** climate warming, distribution, ectothermic, larvae, natural conditions, Pacific lamprey, incipient lethal temperature

## Abstract

In many areas where larval Pacific lampreys currently rear, maximum stream temperatures may approach 27–31 °C during the next 75 years. Whether larval Pacific lampreys in natural conditions can tolerate these temperatures is unknown. To evaluate this ability, we conducted Direct Acute Exposure (DAE) experiments using simulated natural daily temperature (SNT) cycles in the laboratory and occupancy surveys in the Umatilla River (river). When evaluated relative to daily maximum temperatures, after seven days in DAE experiments, 78–100% of larvae survived in 29.1 °C, only larvae acclimated to 26.8 °C survived in 31.0 °C, and no larvae survived in 33.6 °C. Based on daily maximum temperatures, the ultimate upper incipient lethal temperature was estimated to be >30.8 °C using a time to death analysis and >32.0 °C using a percent mortality analysis. Some larvae acclimated to 31.0 °C were also able to survive four consecutive days with a daily maximum temperature of 33.6 °C. In 2018–2020, warm areas of the river experienced maximum temperatures in July and August that ranged from 27.7 to 33.9 °C, while cool areas experienced maximum temperatures <27.7 °C. Before, during and after the period of maximum temperatures each year, larvae occupied both areas. Detection probabilities ranged from 0.83 to 1.00 and were similar for each area and for all survey periods. This work suggests that ectothermic, larval Pacific lampreys in natural environments may be resilient to the water temperatures that are likely to result from climate warming. It is unclear whether relatively high but sublethal temperatures may impact the behavior, and ultimately survival, of larval Pacific lampreys.

## Simple Summary

Climate models suggest that by the year 2100, maximum stream temperatures may approach 27–31 °C in areas where larval Pacific lampreys currently rear. Whether larval Pacific lampreys in natural environments can tolerate these temperatures is unknown. We combined laboratory studies with field surveys to evaluate whether larvae in natural conditions have this ability. In a laboratory setting, some larvae were able to survive days when the maximum temperature reached 33.6 °C. The results also indicated that most larvae may be able to tolerate days when maximum temperatures reach 30.8–32.0 °C. During three years of field surveys, we observed larvae in warm areas where maximum temperatures during the day ranged from 27.7 to 33.9 °C. Our ability to find larvae in areas that were warm, and at times when they were warm, was similar to our ability to find larvae in areas that were relatively cool and at times when areas were not warm. These findings suggest larval Pacific lampreys in natural environments may be resilient to climate warming. However, it is unclear whether warming temperatures may harm larval Pacific lampreys without causing them to die.

## 1. Introduction

For more than three decades, the climate across the globe has been warming at an unusual rate [1,2,3]. Predictions associated with a warming climate include further increases in air temperatures [4]. Although various models exist, each with some degree of uncertainty, it is commonly suggested that global air temperatures in 2100 may be 1–5 °C higher than those from 1900 to 1960 [3,5,6]. Stream temperatures, partly as a function of air temperatures, are a component of climate warming and also predicted to increase during this period [7,8]. Models predicting changes to stream temperatures exist, are variable, and can be significantly impacted by local or regional climatic conditions as well as specific stream conditions [9,10]. However, in general, predictions of warming stream temperatures are often similar to predictions of warming air temperatures. For example, in the western USA, it is commonly suggested that stream temperatures may also increase 1–2 °C, and perhaps as much as 5 °C, by 2080 [9,11,12]. Although of significant concern [13], how deleterious a warming climate may be to aquatic ecosystems and the ectothermic species within remains unclear.

A member of the primitive Agnathans, the holarctic lamprey genera evolved over 450 million years ago, with much of the modern lamprey diversity appearing in the past 20 million years [14]. Lampreys are obligate ectotherms and are directly influenced by ambient water temperature. On a global scale, temperature appears to be a significant environmental variable associated with the distribution of lamprey [15]. Pacific lampreys (*Entosphenus tridentatus*) are native to the north Pacific Ocean and historically ranged throughout the Pacific Rim from Japan to Mexico. They are an anadromous and parasitic species. As such, spawning, embryo incubation, and hatching all occur in freshwater. The larval form appears to be the longest developmental stage in Pacific lamprey, lasting for 2–12 years before a metamorphosis to juveniles and migration to the ocean [16]. During the larval stage, the Pacific lamprey can be found distributed from spawning areas (often in 2nd or 3rd order tributaries) downstream to the boundary between a river basin (sometimes 5th or 6th order streams) and seawater [17,18]. These areas can include coastal streams in a temperate rainforest to high desert streams that are 500–1000 km inland. Thus, the Pacific lamprey can experience a wide range of temperature during its larval stage.

The status of the Pacific lamprey is a conservation concern. The International Union for Conservation of Nature (IUCN) currently lists the Pacific lamprey as a species that is severely fragmented with a decreasing population trend [19]. In the USA, the Pacific lamprey was petitioned for listing under the USA’s Endangered Species Act [20] and are currently managed, in part, under a Conservation Agreement between numerous natural resource agencies [21]. Various threats have been associated with a diminished status of Pacific lamprey [19,21,22,23], including climate warming which has recently received substantial attention [24,25,26,27]. A warming climate may impact numerous life stages of the Pacific lamprey (e.g., [27,28]) as well as the availability and distribution of their prey [22]. Given that the Pacific lamprey likely exists as larva for the majority of its life history, and the relative diversity of environments in which larvae rear, the greatest impact of climate warming may be to this developmental stage.

Larval lamprey from a variety of species can exhibit the ability to tolerate relatively high water temperatures. For example, laboratory experiments have indicated that the larvae of Arctic lamprey (*Lethenteron camtschaticum*), Sea lamprey (*Petromyzon marinus*), northern brook lamprey (*Ichthyomyzon fossor*), American brook lamprey (*Lethenteron appendix*), and European brook lamprey (*Lampetra planeri*) can tolerate water temperatures near 27.0–31.0 °C (e.g., [29,30]). Although information on the ability of larval Pacific Lampreys to tolerate warm water is relatively limited, recent laboratory investigations suggested their ultimate upper incipient lethal temperature (UUILT) is at least 29.2 °C and may exceed 30.2 °C [31]. This laboratory study also found that water temperatures near 27–28 °C may have sublethal impacts on behaviors such as burrowing. However, limited information is available regarding the manner in which warming water temperatures will affect lamprey in natural environments. It has been suggested that climate warming may be a benefit [32] or a detriment [26] to various lamprey species. Arakawa et al. [33] presented evidence supporting the postulate that climate change has influenced the ecology (specifically distribution) of Arctic lamprey, possibly due to rising seawater temperatures [34]. Specific to larval Pacific lamprey, Reid and Goodman [12] found it was distributed in areas of Southern California where modeled mean August temperatures ranged from 10.3 to 25.9 °C and areas of Northern California where actual water temperatures ranged from 18.5 to 32.6 °C. Overall, the impact that a warming climate will ultimately have on the status of ectothermic Pacific lampreys is uncertain [35].

Many of the areas where the Pacific lamprey rears naturally (e.g., [36]) are currently experiencing maximum water temperatures near 26.0 °C [37]. Given predictions that within the next 50 years air and water temperatures may rise 1–5 °C [11], temperatures in areas where some Pacific lampreys exist now may exceed the lethal limit for larvae. Thus, the goal of this investigation was to evaluate the ability of larval Pacific lamprey, an ectothermic species, to tolerate water temperatures that may result from climate warming. To understand more definitively how larval Pacific lampreys may respond to increasing water temperatures, additional studies where they experience relatively high temperatures in natural conditions are needed. This investigation had four specific objectives. Objective 1 was to determine the maximum temperature larval Pacific lampreys can tolerate during a diel cycle. We addressed this objective using SNT cycles in laboratory conditions. We used controlled experiments to evaluate the ultimate upper incipient lethal temperature and trials that were not controlled rigorously to explore the maximum temperature tolerance ability of larvae. Objective 2 was to determine whether larval Pacific lampreys can occupy streams where water temperatures exceeded 27.7 °C. Objective 3 was to determine whether the ability to detect larvae in natural areas where temperatures exceed 27.7 °C is similar to that in areas where temperatures do not exceed 27.7 °C. Objective 4 was to determine whether burrowing into the substrate may provide larvae refuge from temperatures exceeding 27.7 °C.

## 2. Materials and Methods

### 2.1. Laboratory Animals

In 2018, larval Pacific lampreys were collected from Cedar Creek (Washington, DA, USA, 45.93573° N, −122.61902° W). Larvae were captured by electrofishing [38], using a 3 pulse/s current to cause them to leave their burrows [39], after which they were netted and held in an aerated container of water from Cedar Creek. Captured larvae were anesthetized using buffered, tricaine methane sulfonate (MS-222, 50 mg/L, Sigma-Aldrich, St. Louis, MO, USA) and measured (total length, mm). Both Pacific lamprey (*Entosphenus tridentatus)* and western brook lamprey (*Lampetra richardsoni)* occupy Cedar Creek. Larvae longer than 60 mm were identified to genus using caudal characteristics [40]. In total, four events resulted in 120 larval Pacific lampreys being collected and retained for the study (median total length (TL) of larvae during each collection event ranged from 75 to 83 mm; overall range in TL was 61–119 mm). Experimental larvae were transported to a laboratory in aerated coolers. Transit was approximately 45 min.

### 2.2. Direct Acute Exposure Evaluations

Four, Direct Acute Exposure (DAE) experiments [31,41,42] were conducted in 2018. Larvae were collected (*n* = 24–40 per collection) on 15 June, 7 August, 15 August and 22 August. These dates were chosen in an attempt to match stream temperatures to laboratory acclimation temperatures as closely as possible. Water temperature (henceforth temperature) in Cedar Creek during the collection events ranged from 14.5 to 19.4 °C. In general, with minor modifications, each experiment followed a similar protocol as well as used the tank set-up described by Whitesel & Uh [31]. Briefly, each tank contained well water from the Vancouver Trout Hatchery and, during the course of the experiments, was held with a dissolved oxygen range of 4.8–9.1 mg/L and under a simulated natural photoperiod of artificial light. The modifications were as follows: for the acclimation phase of each experiment, individual larvae were randomly assigned and transferred to one of two acclimation tanks (12–20 larvae/tank). For a given experiment, we targeted constant acclimation temperatures of 19 °C, 23 °C, 25 °C, or 27 °C. Although the length of acclimation may influence survival in subsequent tests, the acclimation phase used in this study lasted for 7 d, a common period for DAE experiments [30,31,41]. Once acclimation was complete, a probe was used to agitate larvae from the substrate of an acclimation tank after which they were captured and directly transferred to one of four test tanks (randomly assigned). For each of the four experiments (acclimation temperatures), either three or four (of five possible) temperature regimes were randomly assigned to test tanks. Test temperatures were designed to simulate a natural, diel cycle that would be likely in streams from the Pacific Northwest of the USA [43]. Simulated natural daily temperature cycles were achieved using five submersible heaters, each controlled by a timer and set to a different temperature. For a given test tank, the minimum temperature occurred near 08:00 H and was targeted to be approximately 6 °C colder than the maximum temperature, which occurred near 16:00 H. The pattern of daily temperature generally conformed to the shape of a sine wave with maximum temperatures that targeted either 21 °C, 27 °C, 29 °C, 31 °C, or 33 °C. In the 19 °C acclimation experiment, we placed 9–10 larvae into an SNT cycle that was designed to peak near 27 °C, 29 °C, 31 °C, or 33 °C. In the 23 °C acclimation experiment, we placed 6–7 larvae into an SNT cycle that was designed to peak near 27 °C, 29 °C, or 31 °C. In the 25 °C acclimation experiment, we placed 6–8 larvae into an SNT cycle that was designed to peak near 29 °C, 31 °C, or 33 °C. In the 27 °C acclimation experiment, we placed 8 larvae into an SNT cycle that was designed to peak near 21 °C, 29 °C, or 31 °C and four larvae into an SNT cycle that was designed to peak near 33 °C. During the test phase, larvae were exposed to test temperatures and held for 168 h. Survival was determined at 14 time periods (1, 2, 3, 4, 6, 8, 12, 24. 48, 72, 96, 120, 144, and 168 h). To remove mortalities and ensure that dissolved oxygen remained >4 mg/L, all tanks were monitored at each time interval.

We estimated the upper incipient lethal daily maximum temperature (UILT_DM_), or daily maximum temperature at which 50% of the larvae from a given acclimation temperature survived the 7 d test period. We used an approach based on a constant test temperature [41,44] in which the time to death (TTD) is analyzed to estimate the upper incipient lethal temperature (UILT). Briefly, our analysis involved two steps. Initially, a symmetric sigmoidal regression (Equation (1)),

(1) $$S=d+\frac{a-d}{1+{\left(\frac{T}{c}\right)}^{b}}$$

where S = percent survival, T = the maximum daily temperature, and *a*, *b*, *c*, and *d* = regression coefficients, was used to evaluate the percent survival as a function of time in each test SNT cycle. In many cases, 100% of the larvae survived the entire test period. Whitesel & Uh [31] found that when larvae could not survive a given temperature, 100% generally perished within 7 d and that when larvae could survive a warm temperature (e.g., 27 °C), they did so for up to 30 d. Thus, for the purpose of the regression, if mortality in a given SNT cycle test did not reach 50% after 7 d (e.g., was 100%), we assumed 50% of the larvae would have survived for at least 14 d (2× the length of the test period). Based on the parameters of the regression equations, we then estimated the time to which 50% of the larvae survived (LT50). For a given acclimation temperature, an exponential decay regression was then used to evaluate LT50 as a function of test temperature. The parameters of this regression equation were used to calculate the UILT_DM_. As a complementary approach, we also analyzed percent mortality (PM) after 168 h (7 d) [31]. In our study, after 168 h in the test temperatures, percent survival of each treatment had trials that exceeded and fell below 50%. Thus, to estimate the UILT_DM_ for each acclimation temperature, we also used a symmetric sigmoidal regression to evaluate percent survival as a function of the SNT cycle test temperature. The resulting equation allowed us to calculate the maximum daily temperature at which 50% of the larvae would have been expected to survive. We used the UILT_DM_ values in an attempt to estimate the ultimate UILT_DM_ from each approach. The UUILT is commonly characterized by UILT values that no longer increase with increasing acclimation temperatures [41,44,45] or the maximum temperature to which lamprey can be acclimated [46]. Thus, we attempted to estimate the UUILT_DM_ (7 d) by plotting UILT_DM_ values against acclimation temperature and examining whether there was a temperature where UILT_DM_ values appeared to reach a plateau [47,48,49]. To assist with this process, we regressed UILT_DM_ values on acclimation temperatures, calculated the coefficient of determination (R^2^), and evaluated the significance of the relationship. We also determined whether the slopes of the TTD and PM analyses had overlapping 95% confidence intervals.

### 2.3. Maximum Ability Evaluations

Once the final DAE experiment (27 °C acclimation) was complete and we had determined that larvae could survive for 7 d in the 31 °C test, we then evaluated whether they could survive additional time of daily maximum temperatures near 31 °C. Larvae from the 21 °C (*n* = 8) and 31 °C (*n* = 8) tests of the 27 °C acclimation experiment were returned to their respective test tanks. As in the DAE experiments, we continued to evaluate their survival on a daily basis. Larvae in the 21 °C test tanks essentially served as an experimental control. After 14 d in these conditions with no mortality, to evaluate whether larvae in the 31 °C test tank could survive daily cycles with maximum a temperature near 32 °C, we transferred one larva from the 31 °C test tank to a 32 °C test tank. The 32 °C test tank had an SNT cycle that ranged from approximately 26 to 32 °C. After this larva survived three daily peaks in the 32 °C test tank, and the larvae in the 31 °C test tank (*n* = 7) as well as the 21 °C test tank (*n* = 8) continued to survive, we discontinued the 21 °C test tank control and removed the larvae from the 32 °C test tank. We then evaluated whether larvae that had survived 18 days with maximum temperatures near 31 °C (*n* = 7 remaining in the 31 °C test tank) could survive three daily cycles with maximum temperatures near 33 °C. We transferred two larvae from the 31 °C test tank to a 33 °C test tank with an SNT cycle that ranged from approximately 27–33 °C. Their survival was evaluated on a daily basis. Since both of these larvae survived, we evaluated whether this finding was repeatable. The two larvae in the 33 °C test tank were removed, replaced by two larvae that had survived 21 days of maximum temperatures near 31 °C (*n* = 5), and the survival of those was monitored for three days. This process was repeated a final time using two additional larvae that had survived 25 days of maximum temperatures near 31 °C (*n* = 3).

### 2.4. Natural Distribution Evaluations

**Figure 1:**
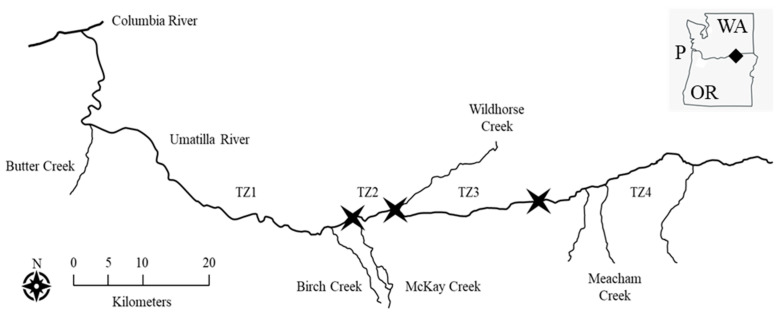
Thermal zones of the Umatilla River (Oregon, USA) used in this study. For the purpose of this study, the river was partitioned into four thermal zones. Thermal Zone 1 (TZ1) was characterized by relatively constant, maximum temperatures near 28.0 °C. Thermal Zone 2 (TZ2) was characterized by relatively constant, maximum temperatures >30.0 °C. Thermal Zone 3 (TZ3) was characterized by maximum temperatures that decreased from approximately 31.0 °C at the downstream end to 27.7 °C at the upstream end. Thermal Zone 4 (TZ4) was characterized by maximum temperatures that decreased from approximately 27.7 °C at the downstream end to <19.9 °C at the upstream end, a distance of approximately 30 river kilometers. The downstream boundary of TZ1 occurs at the confluence with the Columbia River. Due to the hydrogeomorphological characteristics of the watershed, the boundaries (black stars) between TZ1 and TZ2, TZ2 and TZ3, and TZ3 and TZ4 occur at uneven intervals throughout the river. The insert shows the Umatilla River confluence with the Columbia River (black diamond), which then flows downstream approximately 470 river kilometers to the Pacific Ocean (P), bisecting the states of Washington (WA) and Oregon (OR).

To explore the relationship between water temperature and the distribution of larval Pacific lampreys in natural conditions, we used the Umatilla River (Oregon) (henceforth, river) as our study area. Based on historical temperature data (2004–2013), we partitioned the river into four thermal zones (Figure 1). Thermal Zone 2 (TZ2) ranged from the confluence with McKay Creek, upstream approximately 8.5 km (to the boundary of the Umatilla Indian Reservation). TZ2 was characterized by summer maximum temperatures that were expected to exceed 30.0 °C and be relatively constant throughout the zone. Thermal Zone 4 (TZ4) began at a point roughly 20 km upstream of the confluence with Wildhorse Creek, continued upstream approximately 30 km (to the confluence of the North Fork Umatilla River and South Fork Umatilla River). TZ4 was characterized by summer maximum temperatures that were expected to range from <27.7 °C at the downstream end to approximately 19.9 °C at the upstream end, and inversely related to river kilometer and elevation. We considered TZ4 as a control or reference area (relatively cool temperatures resulting in little to no thermal stress) and TZ2 as a treatment or impact area (temperatures approaching or exceeding the putative UUILT for larval Pacific lamprey).

We conducted electrofishing surveys in each thermal zone. Both TZ2 and TZ4 were partitioned into a continuous layer of 50 m long reaches. We identified ordered, random and spatially balanced sample reaches by using a generalized random tessellation stratified (GRTS) approach [50]. Based on the GRTS approach, we selected the 10 highest-ordered reaches in each thermal zone. Based on an assumed detection probability (*d*) of 0.35, this number of reaches (or amount of effort) was chosen so that if lampreys were not detected, the expected probability of occupancy (in a TZ) would be <0.05 [51]. Each sampling event consisted of electrofishing a 50 m reach to determine if larval lampreys were present. Briefly, each reach was sampled by a crew of 2 or 3 people using an AbP-2 backpack electrofisher (Engineering Technical Services, University of Wisconsin, Madison, WI, USA). To stimulate larvae to emerge from burrows, the electrofishing unit was set to deliver 175 volts (DC) at a 25% duty cycle with a 3:1 burst pulse rate [38]. If necessary to capture emergent larvae, the unit was triggered to deliver 30 pulses/s. We spent approximately 30 s/m^2^ within each reach when electrofishing Type I habitat [52] and approximately 5 s/m^2^ when electrofishing Type II and Type III habitats.

To explore the relationship between larval distribution and stream temperature, we implemented a sample design that was a modification of the traditional before-after-control-impact (BACI) approach [53,54]. In 2018, 2019, and 2020, we sampled the river from June to September. Accurately predicting temperature patterns, on a daily or annual basis, is an imperfect process. However, we attempted to implement a sample design relative to the period of maximum temperatures in the summer. We considered this period to be between the first and last daily maximum temperature of at least 27.7 °C in TZ2. We chose this temperature because it appears to reflect a threshold above which Pacific lamprey larvae can tolerate, at which they can survive for extended periods but may be behaviorally compromised [31]. To determine sample times, we monitored temperature using a gauge (PDTO) operated by the U.S. Bureau of Reclamation [55]. To understand the relationship between data from the PDTO gauge and our sample areas, in 2019 we deployed temperature loggers in sample reaches of both TZ2 and TZ4 and evaluated the data using a regression analysis. To understand the relationship between the temperature above and below the surface of the stream bottom, in 2020 we deployed loggers in select reaches of both TZs, either in the water column at the surface of the substrate or buried 4–7 cm below the substrate surface, and we evaluated whether the data had 95% confidence intervals that overlapped. We attempted to sample before, during, and after the period of peak temperatures in TZ2, or as a before-during-after-control-impact (BDACI) sample design. This sample design allowed us to evaluate larval occupancy during each sample period (representing B, D, and A) and in each thermal zone (representing C and I). If a Pacific lamprey larva was captured, the reach (and, thus, TZ) was determined to be occupied and sampling in that reach was terminated. Otherwise, the entire reach was sampled. If no larvae were detected in any reach during a given period, the TZ was considered unoccupied. This sample design also allowed us to evaluate the probability of detecting larvae (*d*) in each TZ during each sample period. We sampled 5–10 reaches in each TZ during each sample period. If a larval Pacific lamprey was detected (*d* = 1.0), then sampling was terminated for that reach. If larvae were detected in at least five reaches of both TZs, sampling for that period (e.g., before) was terminated. This protocol was adopted to maximize the efficiency of sampling effort. If *d* was determined to be 0.5–1.0 in each TZ, additional sampling would not have resulted in a different statistical inference. However, if larval Pacific lampreys were not detected in at least five reaches of both TZs during a given sample period, then all 10 reaches were sampled within each TZ. During each sampling period we compared (i) the occupancy of each TZ and (ii) *d* in each TZ. We also compared whether (iii) occupancy or (iv) *d* within a TZ varied among sampling events. To compare *d*, Fisher’s Exact test was used at a significance level (α) of 0.05. Since we performed nine, planned comparisons (of the 15 total that were possible), a Bonferroni correction (see [56]) was used to adjust α to <0.0056.

## 3. Results

### 3.1. Direct Acute Exposure Evaluations

**Figure 2:**
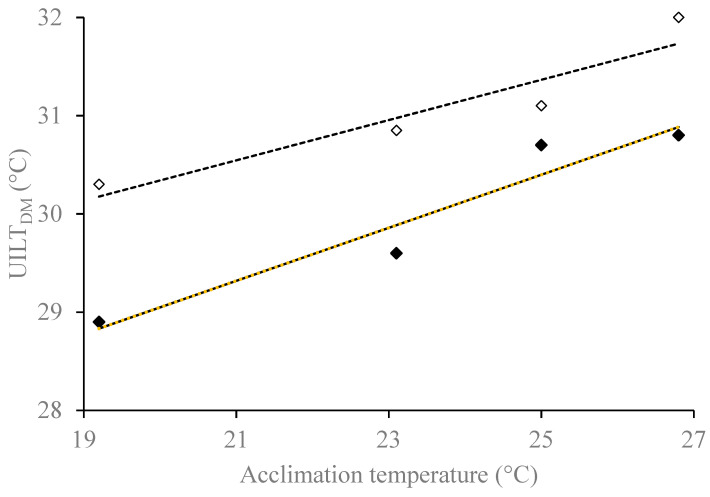
The relationship between acclimation temperature and the upper incipient lethal daily maximum temperature (UILT_DM_). The UILT_DM_ was calculated using both a time to death (TTD, black diamonds) and percent mortality (PM, white diamonds) approach. The TTD analysis (solid line) exhibited a positive, linear relationship with acclimation temperature (slope = 0.270, R^2^ = 0.93, *p* = 0.038). The PM analysis (dashed line) also exhibited a positive, linear relationship with acclimation temperature (slope = 0.204, R^2^ = 0.89, *p* = 0.056). The slopes resulting from the TTD (0.270) and PM (0.204) analyses were similar, exhibiting overlapping 95% confidence intervals. A plateau in the relationship was not evident and the ultimate upper incipient lethal daily maximum temperature could not be determined.

The Direct Acute Exposure (DAE) experiments included four acclimation temperature experiments with actual mean temperatures ranging from 19.2 to 26.8 °C (Table 1). Each acclimation experiment included acute exposure to three or four, simulated natural daily temperature (SNT) cycle test trials with actual maximum daily temperatures that ranged from 21.5 to 33.6 °C. For all combinations of acclimation temperatures from 19.2 to 26.8 °C and tests with peak temperatures that reached 21.5–29.1 °C, 77.8–100.0% of larvae survived for 168 h. In tests with peak temperatures that reached 31.0 °C, 0–100% of larvae survived for 168 h. Larvae in the 31.0 °C tests did not achieve 50% survival after acclimation at temperatures colder than 25.0 °C but did achieve 100% survival after acclimation at 26.8 °C. In all tests with peak temperatures that reached 33.6 °C, 0% of larvae survived for 168 h. In general, survival exhibited a positive relationship to acclimation temperature (particularly evident in the 31 °C tests) and a negative relationship to test temperature. The estimated time to reach 50% mortality (LT50) ranged from 4.9 to 83.6 h. Using the time to death (TTD) approach, the ultimate incipient lethal daily maximum temperature (UILT_DM_) estimates were positively related to acclimation temperature (R^2^ = 0.93, *p* = 0.038) and ranged from 28.9 to 30.8 °C. Using the percent mortality (PM) approach, UILT_DM_ estimates were also positively related to acclimation temperature (R^2^ = 0.89, *p* = 0.056) and ranged from 29.9 to 32.0 °C. Overall, neither the TTD nor PM approach yielded a clear plateau in UILT_DM_ in relation to increasing acclimation temperature (Figure 2). Thus, we could not estimate a specific ultimate upper incipient lethal daily maximum temperature (UUILT_DM_). However, since UILT_DM_ continued to increase with acclimation temperature, we were able to use both the TTD and PM approach to estimate an UUILT_DM_ of at least 30.8 °C and 32.0 °C, respectively. The slopes of each analysis were similar (0.270 and 0.204, respectively) with overlapping 95% CIs. The total length of larvae used in DAE trials ranged from 61 to 119 mm and, for a given acclimation temperature, they were randomly assigned to each test group.

### 3.2. Maximum Ability Evaluations

All larvae that were acclimated to 26.8 °C survived for 14 days after a transfer to an SNT cycle that had a daily maximum of 21.5 °C (*n* = 8). All larvae (100%) that were acclimated to 26.8 °C also survived for 14 days (*n* = 8), 18 days (*n* = 7), 21 days (*n* = 5), or 25 days (*n* = 3) after a transfer to an SNT cycle that had a daily maximum of 31.0 °C. One larva (100%) that was acclimated to 26.8 °C and then survived for 14 days in an SNT cycle that had a daily maximum of 31.0 °C was also able to survive three days after a transfer to an SNT cycle that had a daily maximum of 32.0 °C. Most larvae (83.3%) that were acclimated to 26.8 °C and then survived for 18 days (*n* = 1), 21 days (*n* = 2), or 25 days (*n* = 2) in an SNT cycle that had a daily maximum of 31.0 °C were also able to survive three days after a transfer to an SNT cycle that had a daily maximum of 33.6 °C. One larva (16.7%), that was acclimated to 26.8 °C and then survived for 18 days in an SNT cycle that had a daily maximum of 31.0 °C, survived only one day after a transfer to an SNT cycle that had a daily maximum of 33.6 °C.

### 3.3. Natural Distribution Evaluations

**Figure 3:**
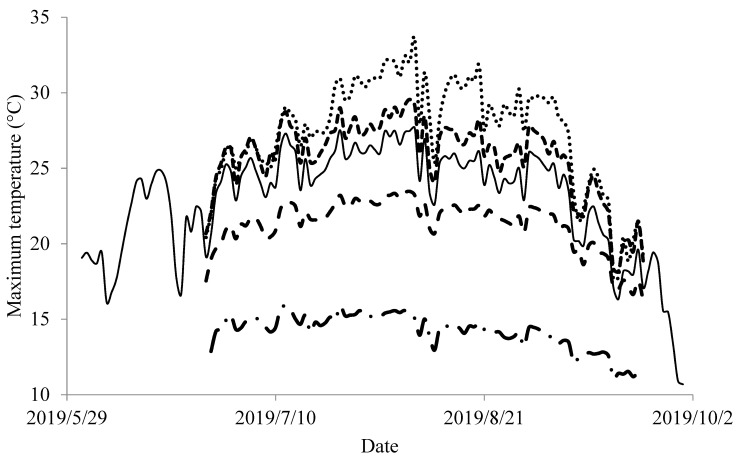
Daily temperatures during the study period in 2019. Actual maximum daily temperatures were recorded at the most downstream (stippled gray line) and most upstream (thick dashed gray line) reach of Thermal Zone 2 (TZ2). Actual maximum daily temperatures were also recorded at the most downstream reach (thin dashed gray line) and most upstream reach (dashed, stippled gray line) of Thermal Zone 4 (TZ4). In addition, monitoring efforts not directly related to this study recorded actual maximum temperatures (solid black line) at a gauge (PDTO) near Pendleton, Oregon (USA). Regression analyses indicated that the temperatures recorded at PDTO exhibited a significant, linear relationship to the most upstream reach in TZ2 (R^2^ = 0.989, *p* < 0.001) and the most downstream reach in TZ4 (R^2^ = 0.949, *p* < 0.001).

During the 2019 study period, daily maximum temperatures recorded at the PDTO gauge in TZ2 ranged from 16.3 to 27.7 °C (Figure 3). During this period, daily maximum temperatures recorded at the most downstream reach of TZ2, most upstream reach of TZ2, most downstream reach of TZ4, and most upstream reach of TZ4 ranged from 17.7 to 33.6 °C, 18.1–29.6 °C, 16.2–23.4 °C, and 11.1–15.9 °C, respectively. The temperatures recorded at PDTO exhibited a significant, linear relationship to those in each reach of TZ2 and TZ4 (*p* < 0.001). In particular, there was a strong relationship between PDTO and both the most upstream reach in TZ2 (R^2^ = 0.989) and the most downstream reach in TZ4 (R^2^ = 0.949). The period during which peak temperatures (>27.7 °C) occurred in TZ2 was from 11 July to 30 August. The overall maximum temperature was 33.6 °C in TZ2 and 23.5 °C in TZ4 (where it did not exceed 27.7 °C). We electrofished before this period (25–26 June), during this period (21 August), and after this period (23–24 September). We electrofished five reaches in each TZ during each sample period. Larval Pacific lampreys occupied TZ2 and TZ4 before, during, and after the period of peak temperatures. For both TZs and each sample period, patterns of occupancy were similar. For both TZ2 and TZ4 before, during, and after the period of peak temperatures, *d* = 1.00. For both TZs and each sample period, *d* was similar (Fisher Exact, DF = 1, *p* = 1.00).

During the 2018 study period, daily maximum temperatures recorded at the PDTO gauge in TZ2 ranged from 14.8 to 29.2 °C. Based on the relationship between temperatures recorded at PDTO and those throughout TZ2 and TZ4 (see 2019 results), the period during which peak temperatures (>27.7 °C) occurred in TZ2 was from 20 June to 18 August. We electrofished before this period (18–19 June), during this period (26 July), and after this period (27–28 August). Given the actual temperature pattern, this design also allowed us to sample before, during and after the period between the first and last daily maximum temperature of at least 30.1 °C in TZ2 (14 July–9 August). The overall maximum temperature was 33.9 °C in TZ2 and 24.4 °C in TZ4 (where it did not exceed 27.7 °C). We electrofished five or six reaches in each TZ during each sample period. Larval Pacific lampreys occupied TZ2 and TZ4 before, during, and after the period of peak temperatures. For both TZs and each sample period, patterns of occupancy were similar. For both TZ2 and TZ4 before, during, and after the period of peak temperatures, *d* ranged from 0.83 to 1.00. For both TZs and each sample period, *d* was similar (Fisher’s Exact test, DF = 1, *p* = 1.00).

During the 2020 study period, daily maximum temperatures recorded at the PDTO gauge in TZ2 ranged from 15.8 to 29.5 °C. Based on the relationship between temperatures recorded at PDTO and those throughout TZ2 and TZ4 (see 2019 results), the period during which peak temperatures (>27.7 °C) occurred in TZ2 was from 16 July to 22 August. The overall maximum temperature was 34.4 °C in TZ2 and 24.6 °C in TZ4 (where it did not exceed 27.7 °C). We electrofished before this period (25–26 June), during this period (13 August), and after this period (23 September). Given the actual temperature pattern, this design also allowed us to sample before, during, and after the period between the first and last daily maximum temperature of at least 29.5 °C in TZ2 (20 July–18 August). We electrofished five or six reaches in each TZ during each sample period. Larval Pacific lampreys occupied TZ2 and TZ4 before, during, and after the period of peak temperatures. For both TZs and each sample period, patterns of occupancy were similar. For both TZ2 and TZ4 before, during, and after the period of peak temperatures, *d* ranged from 0.83 to 1.00. For both TZs and each sample period, *d* was similar (Fisher’s Exact test, DF = 1, *p* = 1.00).

**Figure 4:**
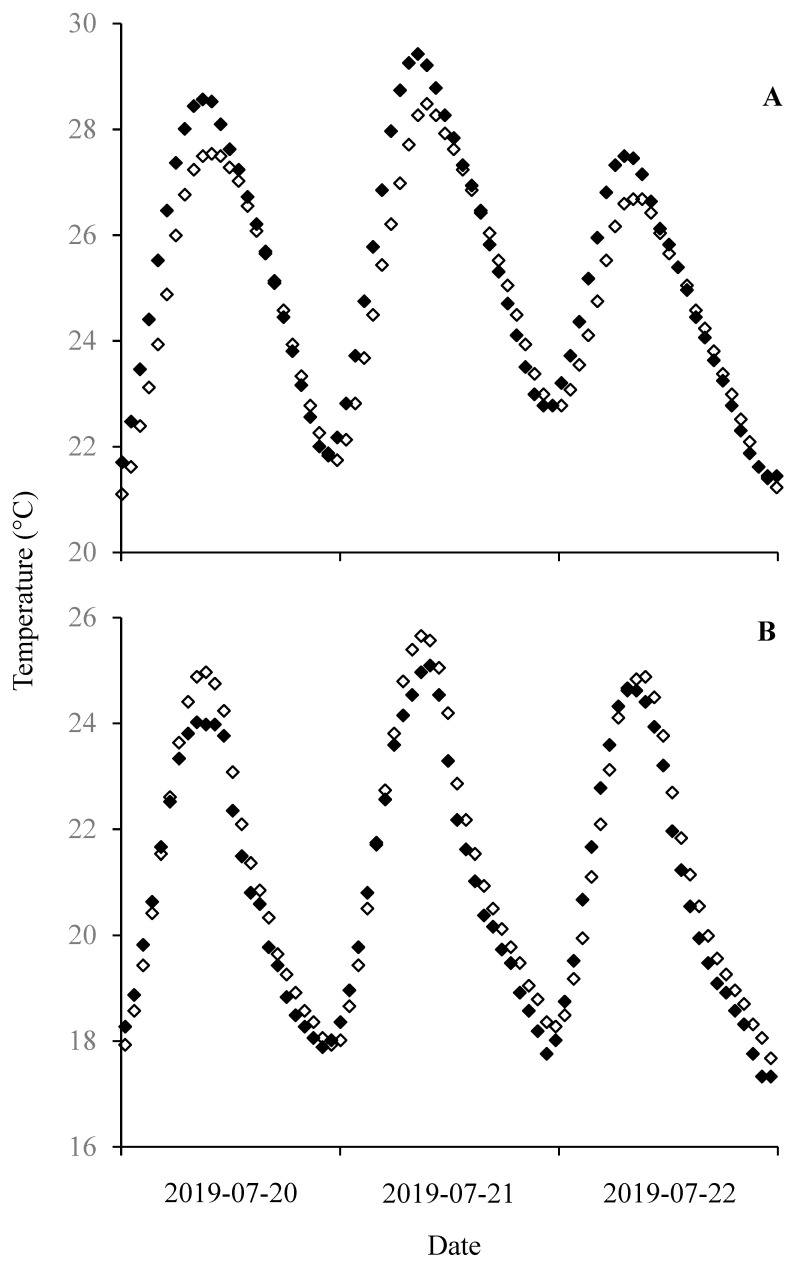
A comparison of diel temperature cycles in the Umatilla River, above (black diamonds) and below (white diamonds) the substrate surface. The upper panel (**A**) represents the most downstream reach in Thermal Zone 2 (TZ2) and the lower panel (**B**) represents the most downstream reach in Thermal Zone 4 (TZ4). Loggers, held in opaque tubing and either placed in the water column on the surface of the substrate or buried 4–7 cm below the substrate surface at the same location, recorded temperatures for the duration of the study. For the 2019 study period, when compared to those in the water column, maximum temperatures below the substrate surface were significantly cooler in TZ2 (mean = −0.51 °C, 95% confidence interval = 0.10) and similar in TZ4 (mean = −0.13 °C, 95% confidence interval = 0.16).

During 2020, in the most downstream reach of TZ2, the mean temperature below the substrate surface was significantly colder than that in the water column near the substrate surface (mean = −0.51 °C, 95% CI = 0.10). In general, temperatures below the substrate surface changed in a pattern that was similar to that above the substrate (Figure 4). However, subtle differences were evident, with temperatures below the substrate beginning to increase later in the day and increasing more slowly than those above the substrate. From 1 June to 31 July, temperatures below the substrate surface exceeded 27.7 °C on three days, 1.3% of the total study period, with a maximum temperature of 28.7 °C. In the most downstream (warmest) reach of TZ4, the mean temperature below the substrate surface was similar to that in the water column (−0.13 °C, 95% CI = 0.16). Temperatures below the substrate surface also changed in a pattern similar to that above the substrate (Figure 4).

## 4. Discussion

Larval Pacific lampreys can tolerate days that have maximum temperatures exceeding 33 °C. This was most apparent in laboratory conditions where larvae were able to survive for 3 days while they experienced diel peaks of 33.6 °C. In addition, this was consistent with UUILT_DM_ estimates for these larvae of at least 30.2–32.0 °C. This finding is among some of the warmest tolerance levels reported for larval lamprey, but not unprecedented. Smirnov et al. [57] estimated the upper lethal temperature for larval river lampreys (*Lampetra fluviatilis*) in Russia to be 34.3–34.5 °C. The ability of larval Pacific lampreys to tolerate temperatures > 33 °C is also relatively great when compared to many sympatric fish species. For example, the upper thermal tolerance ability for *Salvelinus confluentus* [58] and *Oncorhynchus tshawytscha* [41,59] ranges from 21 to 25 °C and is unlikely to exceed 30 °C (reviewed by [60,61]). Unlike many of the anadromous species with which they are sympatric, the Pacific lamprey does not appear to express natal homing during spawning [62]. Given this life history, along with its distribution across a vast geography that exhibits a wide range of temperatures, it may not be surprising that the Pacific lamprey has developed an ability to tolerate the warmest temperatures they would likely encounter throughout their range. The ability to tolerate temperatures >33 °C that was demonstrated by larval Pacific lampreys is surpassed by some ectothermic fish species that can be found in freshwater environments of North America but are not from the order Petromyzontiformes. For example, *Fundulus heteroclitus* and *Ictalurus punctatus* have been shown to have critical thermal maximum temperatures exceeding 40 °C (reviewed in [44]). It is important to note the number of larvae we were able to test for maximum ability was limited (*n* = 6). Furthermore, while we attempted to determine the ability of larval Pacific lampreys to tolerate relatively warm temperatures in natural (or simulated-natural) conditions, it is possible there are circumstances where they cannot tolerate temperatures as warm as 33 °C. For larvae to tolerate 33 °C in simulated-natural conditions, it was necessary for them to be acclimated to 31 °C and the duration of the daily maximum temperature did not exceed 120 min. Finally, for larvae to tolerate 33 °C in natural conditions, it was unclear when they entered areas of high temperatures or how long they occupied those areas. Although all circumstances that would allow larval Pacific lampreys to tolerate temperatures > 33 °C are unclear and likely variable, it is clear that they have this ability.

Larval Pacific lampreys can tolerate multiple, consecutive days where maximum temperatures reach 31 °C. Several lines of evidence support this claim. Larvae were able to survive for as many as 18 days while they experienced diel cycles in which the daily maximum temperature was 31.0 °C. Furthermore, minimum estimates of the UUILT_DM_ ranged from 30.8 to 32.0 °C. Although not perfectly comparable to traditional measures of UUILT that are derived from constant test temperatures, the level of temperature tolerance we observed is generally consistent with literature on other lamprey species. For example, using experiments with constant test temperatures, Potter and Beamish [29] estimated the UUILT for multiple species to range from 29.2 to 31.4 °C. Similarly, Arakawa and Yanai [30] estimated the UUILT for Arctic lampreys (*Lethenteron camtschaticum*) to be 29.3 °C. In addition, using experiments with increasing test temperatures, Golovanov et al. [63] estimated the critical thermal maximum (CTM) and upper lethal temperature for European river lampreys (*Lampetra fluviatilis*) to be 29.0 °C and 30.8 °C, respectively. The length of time larvae can tolerate such conditions has not been well studied. Specifically, reports from laboratory studies evaluating lampreys exposed to test temperatures for >7 d are uncommon. While the ability of larvae to tolerate daily maximum temperatures as warm as 31 °C for 11–18 days may be a relatively novel finding, it integrates well with what is known. Whitesel and Uh [31] reported that larval Pacific lampreys persisted in a constant temperature of 27.7 °C for 30 days. Similarly, Arakawa and Yanai [30] reared larval Arctic lampreys in constant temperatures for 30 d and reported inhibited growth and poor survival only after temperatures reached or exceeded 28 °C. Furthermore, the ability of larval Pacific lampreys to tolerate daily peak temperatures of 31 °C for 11–18 days is within or exceeds the conditions they would likely experience naturally. On an annual basis, the period of maximum temperatures experienced by larval Pacific across their range generally does not last longer than approximately 14 days [64].

Larval Pacific lampreys in natural environments are able to occupy, and presumably persist in, areas that reach temperatures from 27.7 to 33.6 °C. This was evidenced by our ability to detect larvae in areas of the river that reached this temperature range, as well as to detect them before, during, and after the period of these temperatures. Further, our ability to detect larvae in these areas was similar to that in areas of the river that did not exceed 27.7 °C. This finding is consistent with results from clinical work reported here as well as by Whitesel and Uh [31]. The utility of distribution studies in the natural environment to compliment clinical evaluations of thermal capability has been recognized by numerous researchers studying a variety of species [65,66,67,68]. While we are unaware of any intensive and direct applications of this approach with larval Pacific lamprey, our findings are consistent with the work of Reid and Goodman [12] who performed an extensive evaluation of the natural distribution of larval Pacific lampreys in the southern portion of their range, including all of Southern California (USA) and northern Mexico. They explored the association between several scenarios of modeled stream temperatures in August and various sources of information indicating whether stream reaches were currently occupied by lampreys. For validation purposes, they also surveyed and measured temperatures at eight sites in northern California (USA). Reid and Goodman found larval Pacific lampreys occupied areas where the actual water temperature was >32 °C and the modeled water temperature was as high as 25.9 °C. Thus, it may not be uncommon for larval Pacific lampreys to occupy areas where stream temperatures reach 30–33 °C.

While evidence from stream surveys suggests larval Pacific lampreys can tolerate relatively warm temperatures, there are several alternatives to this interpretation. It is unlikely that we were detecting the same individual larva before, during, and after the period of maximum temperatures. Thus, an individual larva may only have occupied TZ2 on the day we surveyed. Larvae were not tagged, and it is unclear if and how they moved within and between thermal zones. Larvae that entered TZ2 could have left immediately or remained there for the duration of the study as well as died immediately or survived for the duration of the study. However, given that each thermal zone was occupied during all sampling periods, if larvae were constantly emigrating from or dying in TZ2, it is apparent that larvae from other (cooler) thermal zones were constantly immigrating to TZ2. Larval Pacific lampreys are not considered to be active and strong swimmers but relatively sessile and quickly exhausted by activity [69]. Given these characteristics, it may be reasonable to assume their maximum migration rate would be roughly equal to stream flow (or movement of a water particle). Typical water velocity during this study [43] applied to the length of TZ2 suggests that a larva entering TZ2 from the upstream end would likely be in TZ2 for a minimum of 5 h. Since all of the larvae we captured were burrowed and not in the water column, it is likely they were moving through TZ2 at a slower rate than stream velocity. In addition, during a related study in which we tagged larvae in TZ2, some were recaptured in TZ2 approximately 30 d later. Taken as a whole, in TZ2, it is reasonable to presume that all larvae which enter TZ2 likely persist there for a minimum of 5 h, likely longer, and some for more than 30 d. Overall, occupancy and temperature surveys in the river support the notion that larval Pacific lampreys in natural environments can tolerate and persist in temperatures that reach 33.6 °C for periods up to 120 min.

The ecological status of larval Pacific lampreys appeared to be independent of the current thermal conditions they would likely encounter naturally. This included relatively warm water temperatures with daily maximum temperatures between 27.7 and 33.6 °C. This claim is supported by the finding that larvae occupied both TZ2 and TZ4, the warmest and coolest areas of the river, respectively. Furthermore, equal detection probabilities were observed before, during, and after the periods of maximum temperatures, in both TZ2 and TZ4. While the specific relationship between the detection probability for and abundance of a species is often unknown [70], understanding detection probability is a necessary component of properly evaluating abundance [71]. In particular, information on detection probability can potentially be useful as a surrogate for abundance [72] and measures of population status [73]. The notion that relatively warm thermal conditions may not be restrictive to the natural abundance or distribution of larval Pacific lampreys is consistent with the work of Reid and Goodman [12] who predicted or found that multiple size (presumably age) classes of larval Pacific lampreys occupied temperatures ranging from 25.9 to 32.6 °C. Alternatively, although they may have the ability to distribute throughout and be relatively abundant in areas of warm temperatures, these thermal conditions may not be optimal for larval Pacific lamprey. Meeuwig et al. [28] reported that survival after hatching for both Pacific lamprey and western brook lamprey was greatest near 18 °C. Investigations on larval sea lamprey found them to voluntarily occupy and possibly prefer temperatures from 10 to 19 °C [46]. Similarly, laboratory studies found that larval sea lamprey may prefer temperatures from 15 to 23 °C and function optimally from 19 to 21 °C [74,75]. The temperature larvae can tolerate is unlikely to be the same as the temperature at which they function best or prefer [46]. Currently, however, any such differences do not appear to limit the distribution and abundance of larval Pacific lampreys in natural conditions.

Burrowing in the substrate may provide larval Pacific lampreys a mechanism to obtain some refuge from relatively warm temperatures. In the Umatilla River, this was demonstrated by peak temperatures below the substrate infrequently reaching or exceeding 27.7 °C and only reaching a maximum of 28.7 °C. Furthermore, in the warmest areas, mean temperatures 4–7 cm below the surface of the substrate were >0.5 °C cooler than mean temperatures in the water column at the surface of the substrate. A potentially cooler temperature below the surface of the substrate is not a novel finding. For example, Reid and Goodman [12] observed mean in situ substrate temperature that averaged −0.8 °C when compared to overlying water temperature. Furthermore, fish seeking and using cool areas as refuges from warm conditions has been observed previously. For example, rainbow trout (*Oncorhynchus mykiss*) in arid regions have been shown to use cool areas, potentially allowing them to persist in warm conditions that would otherwise be suboptimal or lethal [76]. However, while temperature in the substrate may generally be cooler than that in the water column (e.g., [12]), stream hydraulics [77] and local conditions [78] can have a significant influence on this temperature. In addition, while temperatures in the substrate may be cooler than those in the water column, they may still exceed lethal or optimal values. Thus, even when burrowed in the substrate, larvae may not find an absolute refuge from relatively warm temperatures. Overall, the importance to larval Pacific lampreys of temperatures in the substrate is not well understood.

To our knowledge, this is one of the first studies to use SNT cycles in experiments on incipient lethal temperatures. This experimental adjustment may be significant to the interpretation of our results. Traditionally, UUILT is defined as the warmest temperature at which 50% of a population could survive indefinitely [47,48]. In contrast to a traditional UUILT, critical thermal maximum (CTM) values are generally higher and represent the maximum temperature that an organism can survive [68,79]. In theory, organisms can tolerate warm temperatures that are below their UUILT; as temperature increases from UUILT to CTM values, there is an inverse relationship with the time an organism can resist the temperature, and at temperatures higher than their CTM an organism perishes almost instantaneously [49]. While intended to represent the natural ability of an organism, both UUILT and CTM are derived from clinical experiments using unnatural test conditions. In this investigation, we modified our experiments to test diel cycles that simulated natural temperatures. In addition, it has been suggested that there may be a little to no acclimation response ratio in larval lampreys (see [16]), potentially making the utility of UUILT studies uncertain. However, consistent with previous studies on the larvae of Arctic [30] and Pacific [31] lamprey, we also found a clear relationship between acclimation temperature and UUILT_DP_. The appropriate temperature metric is also an important consideration. Particularly in cases where temperatures vary, it has been suggested that integrated values (such as daily heat units or degree-days) may be most useful (e.g., [28,80]). Since a change in body temperature is a function of fish size [81], we expected larval temperature to closely mimic that of their environment. Thus, to try and best understand what might be happening under natural conditions, we expressed our findings as a function of the maximum temperature value on a given day. Given these considerations, although it may be more similar to a CTM value than to a traditional UUILT value for larval Pacific lamprey, the UUILT_DP_ that we determined (at least 30.8–32.0 °C) appears to have merit.

## 5. Conclusions

The ultimate impact that a warming climate will have on the status of ectothermic Pacific lampreys is uncertain. However, given that Pacific lampreys likely exist as larvae for the majority of their life history [16], the greatest impact of climate warming may be to this developmental stage. When considering predictions across their range, larval Pacific lampreys may be resilient to maximum warming scenarios [43,82]. We observed larvae tolerating daily maximum temperatures >30.0 °C for two consecutive days in the river and three days in the laboratory. We cannot rule out that larvae can tolerate more than three days with peak temperatures >33 °C. This is consistent with the suggestion that climate warming may not be a threat to all ectothermic fish species [83]. The mechanisms that allow larval Pacific lampreys to tolerate warm temperatures are not clear. As an ectothermic species that does not regulate internal temperature, presumably the internal milieu of a larva reaches temperatures that approximate the ambient temperature. Thus, they appear to have some ability for cellular processes to function at temperatures near 33 °C. While they can survive these temperatures, it is unclear how resources are being used to protect against dysfunction. For example, the cellular production of heat shock proteins (an energy demanding process) is induced following temperature elevations of 13–16 °C for sea lampreys and 16–20 °C for brook lampreys (*Lampetra appendix*) [84]. Larval Pacific lampreys may also use behavioral strategies to mitigate the impacts of relatively high temperatures [12]. Altered physiological, behavioral, or morphological processes may represent important sublethal effects from a warming climate that are significant but poorly understood. The work we present suggests that larval Pacific lampreys in natural environments may be resilient to the summer water temperatures that are likely to result from climate warming. However, to understand more clearly how larval Pacific lampreys will respond to climate warming, additional studies on sublethal impacts in natural conditions are necessary. Furthermore, to understand how the species of Pacific lamprey will respond to climate warming, the adult life stage (which may also persist for approximately a year in similar freshwater areas as larvae) as well as possible impacts of warmer winter temperatures should also be evaluated.

## Figures and Tables

**Table 1:** Summary of four Direct Acute Exposure (DAE) experiments. For each experiment, larval Pacific lampreys were acclimated to a given temperature for seven days then placed in one of four test temperature trials. To mimic diel patterns, simulated natural daily temperature (SNT) cycles were used for test trials. Survival indicates the percent of larvae (*n* = associated sample size) that were alive after 168 h in a test temperature trial. LT50 is the estimated time to which 50% of the larvae survived in a test temperature trial.

Experiment	Acclimation Temperature (°C) ^a^	Test Temperature (°C) for SNT Cycles ^b^	Survival (% [*n*])	LT50 (h)
1	19.2	21.2–27.0	77.8 [9]	-
		22.4–29.1	90.0 [10]	-
		25.1–31.0	0.0 [10]	11.2
		27.0–33.6	0.0 [10]	4.9
2	23.1	21.2–27.0	100.0 [6]	-
		22.4–29.1	100.0 [7]	-
		25.1–31.0	16.7 [6]	83.6
3	25.0	22.4–29.1	85.7 [7]	-
		25.1–31.0	50.0 [8]	169.3
		27.0–33.6	0.0 [6]	9.8
4	26.8	17.3–21.5	100.0 [8]	-
		22.4–29.1	100.0 [8]	-
		25.1–31.0	100.0 [8]	-
		27.0–33.6	0.0 [4]	12.3

^a^ Actual mean temperature during acclimation. ^b^ Actual temperature range during the test trials. - Mortality did not reach 50 (e.g., survival was 100%), and LT50 was not estimated directly.

## Data Availability

Data and material are stored by the U.S. government and under the OPEN Government Data Act, upon request, can be made available to the public.

## References

[1] Intergovernmental Panel on Climate Change (IPCC) (1990). The IPCC scientific assessment. Contribution of Working Group I to the First Assessment Report of the Intergovernmental Panel on Climate Change.

[2] Houghton J.T., Jenkins G.J., Ephraums J.J. (1990). Climate change: The IPCC scientific assessment. Am. Sci..

[3] Lee H., Romero J., Intergovernmental Panel on Climate Change (IPCC) (2023). Climate Change 2023: Synthesis Report. Contribution of Working Groups I, II and III to the Sixth Assessment Report of the Intergovernmental Panel on Climate Change.

[4] Rosenzweig C. (1989). Global climate change: Predictions and observations. Am. J. Agric. Econ..

[5] Meehl G.A., Stocker T.F., Collins W.D., Friedlingstein P., Gaye A.T., Gregory J.M., Zhao Z.C., Solomon S., Qin D., Manning M., Chen Z., Marquis M., Averyt K.B., Tignor M., Miller H.L. (2007). Global climate projections. Climate Change 2007: The Physical Science Basis, Contribution of: Working Group I to the Fourth Assessment Report of the Intergovernmental Panel on Climate Change.

[6] Wuebbles D.J., Fahey D.W., Hibbard K.A., DeAngelo B., Doherty S., Hayhoe K., Horton R., Kossin J.P., Taylor P.C., Waple A.M., Wuebbles D.J., Fahey D.W., Hibbard K.A., Dokken D.J., Stewart B.C., Maycock T.K. (2017). Climate science special report: Fourth national climate assessment, Executive summary.

[7] Isaak D.J., Wollrab S., Horan D., Chandler G. (2012). Climate change effects on stream and river temperatures across the northwest US from 1980–2009 and implications for salmonid fishes. Clim. Change.

[8] Arismendi I., Safeeq M., Dunham J.B., Johnson S.L. (2014). Can air temperature be used to project influences of climate change on stream temperature?. Environ. Res. Lett..

[9] Isaak D.J., Wenger S.J., Peterson E.E., Ver Hoef J.M., Nagel D.E., Luce C.H., Hostetler S.W., Dunham J.B., Roper B.B., Wollrab S.P. (2017). The NorWeST summer stream temperature model and scenarios for the western US: A crowd-sourced database and new geospatial tools foster a user community and predict broad climate warming of rivers and streams. Water Resour. Res..

[10] Isaak D.J., Luce C.H., Horan D.L., Chandler G.L., Wollrab S.P., Dubois W.B., Nagel D.E. (2020). Thermal regimes of perennial rivers and streams in the western United States. J. Am. Water Resour. Assoc..

[11] Wu H., Kimball J.S., Elsner M.M., Mantua N., Adler R.F., Stanford J. (2012). Projected climate change impacts on the hydrology and temperature of Pacific Northwest rivers. Water Resour. Res..

[12] Reid S.B., Goodman D.H. (2024). Exploring thermal conditions occupied by Lampreys (Petromyzontidae) in California and Northern Baja California: Current environment and implications for future scenarios. Environ. Biol. Fish..

[13] Poff N.L., Brinson M.M., Day J.W. (2002). Aquatic Ecosystems and Global Climate Change.

[14] Brownstein C.D., Near T.J. (2023). Phylogenetics and the Cenozoic radiation of lampreys. Cur. Biol..

[15] Ferreira A.F., Quintella B.R., Maia C., Mateus C.S., Alexandre C.M., Capinha C., Almeida P.R. (2013). Influence of macrohabitat preferences on the distribution of European brook and river lampreys: Implications for conservation and management. Biol. Conserv..

[16] Dawson H.A., Potts D.D., Maguffee A.C., O’Connor L.M. (2015). Feasibility of passive integrated transponder technology to study in situ movements of larval sea lamprey. J. Fish. Wildl. Manag..

[17] Jolley J.C., Silver G.S., Whitesel T.A. (2012). Occupancy and detection of larval Pacific lampreys and Lampetra spp. in a large river: The Lower Willamette River. Trans. Am. Fish. Soc..

[18] Blanchard M.R., Harris J.E., Skalicky J.J., Silver G.S., Jolley J.C. (2023). Patterns in distribution and density of larval lampreys in the main-stem Columbia River, Washington–Oregon. N. Am. J. Fish. Manag..

[19] The International Union for Conservation of Nature (IUCN) (2020). Red List of Species. https://www.iucnredlist.org/species/62225/18232572.

[20] Wicks-Arshack A., Dunkle M., Matsaw S., Caudill C. (2018). An ecological, cultural and legal review of Pacific lamprey in the Columbia River basin. Idaho L. Rev..

[21] Pacific Lamprey Conservation Initiative (PLCI) Conservation Agreement. https://www.pacificlamprey.org/wp-content/uploads/2022/03/2-PLCI-2022-Conservation-Agreement-FAQ.pdf.

[22] Almeida P.R., Arakawa H., Aronsuu K., Baker C., Blair S.R., Beaulaton L., Belo A.F., Kitson J., Kucheryavyy A., Kynard B. (2021). Lamprey fisheries: History, trends and management. J. Great Lakes Res..

[23] McLaughlin R., Adams J.V., Almeida P.R., Barber J., Burkett D.P., Docker M.F., Johnson N.S., Moser M.L., Muir A.M., Pereira D.L. (2021). Foreword: Control and conservation of lampreys beyond 2020-Proceedings from the 3rd Sea Lamprey International Symposium (SLIS III). J. Great Lakes Res..

[24] Reid S.B., Goodman D.H. (2016). Pacific lamprey in coastal drainages of California: Occupancy patterns and contraction of the southern range. Trans. Am. Fish. Soc..

[25] Wang C., Schaller H. (2015). Conserving Pacific lamprey through collaborative efforts. Fisheries.

[26] Wang C.J., Schaller H.A., Coates K.C., Hayes M.C., Rose R.K. (2020). Climate change vulnerability assessment for Pacific lamprey in rivers of the western United States. J. Freshw. Ecol..

[27] Clemens B.J. (2022). Warmwater temperatures (≥20 °C) as a threat to Pacific lamprey: Implications of climate change. J. Fish Wildl. Manag..

[28] Meeuwig M.H., Bayer J.M., Seelye J.G. (2005). Effects of temperature on survival and development of early life stage Pacific and western brook lampreys. Trans. Am. Fish. Soc..

[29] Potter I.C., Beamish F.W.H. (1975). Lethal temperatures in ammocoetes of four species of lampreys. Acta Zool..

[30] Arakawa H., Yanai S. (2021). Upper thermal tolerance of larval Arctic lamprey (*Lethenteron camtschaticum*). Ichthyol. Res..

[31] Whitesel T.A., Uh C.T. (2023). Upper temperature limit of larval Pacific lamprey Entosphenus tridentatus: Implications for conservation in a warming climate. Environ. Biol. Fishes.

[32] Lennox R.J., Bravener G.A., Lin H.Y., Madenjian C.P., Muir A.M., Remucal C.K., Robinson K.F., Rous A.M., Siefkes M.J., Wilkie M.P. (2020). Potential changes to the biology and challenges to the management of invasive sea lamprey Petromyzon marinus in the Laurentian Great Lakes due to climate change. Glob. Change Biol..

[33] Arakawa H., Kishi D., Yanai S. (2021). Historical distribution of Arctic lamprey *Lethenteron camtschaticum* in Japanese rivers and its change estimated from fishery statistics and fishers’ local ecological knowledge. Fisher. Sci..

[34] Yamazaki Y., Yokoyama R., Nagai T., Goto A. (2011). Formation of a fluvial non-parasitic population of *Lethenteron camtschaticum* as the first step in petromyzontid speciation. J. Fish. Biol..

[35] Wang C.J., Hudson J.M., Lassalle G., Whitesel T.A. (2021). Impacts of a changing climate on native lamprey species: From physiology to ecosystem services. J. Great Lakes Res..

[36] Clemens B.J., Beamish R.J., Coates K.C., Docker M.F., Dunham J.B., Gray A.E., Hess J.E., Jolley J.C., Lampman R.T., McIlraith B.J. (2017). Conservation challenges and research needs for Pacific lamprey in the Columbia River basin. Fisheries.

[37] United States Geological Survey (USGS) Current Water Data. https://waterdata.usgs.gov/nwis/current/?type=quality.

[38] Stone J., Barndt S. (2005). Spatial Distribution and Habitat Use of Pacific Lamprey (*Lampetra tridentata*) Ammocoetes in a Western Washington Stream. J. Freshw. Ecol..

[39] Weisser J.W., Klar G.T., Cowx I. (1990). Electric fishing for sea lampreys (*Petromyzon marinus*) in the Great Lakes region of North America. Developments in Electric Fishing.

[40] Docker M.F., Silver G.S., Jolley J.C., Spice E.K. (2016). Simple genetic assay distinguishes lamprey genera Entosphenus and Lampetra: Comparison with existing genetic and morphological identification methods. N. Am. J. Fish. Manag..

[41] Brett J.R. (1952). Temperature tolerance in young Pacific salmon, genus Oncorhynchus. J. Fish. Board Can..

[42] Kaya C.M. (1978). Thermal resistance of rainbow trout from a permanently heated stream, and of two hatchery strains. Prog. Fish-Cult..

[43] United States Geological Survey (USGS) Stream Flow Data in the USA. https://waterdata.usgs.gov/wa/nwis/current?type=qw&PARAmeter_cds=STATION_NM,DATETIME,00010,00011.

[44] Beitinger T.L., Bennett W.A., McCauley R.W. (2000). Temperature tolerances of North American freshwater fishes exposed to dynamic changes in temperature. Environ. Biol. Fish..

[45] Fry F.E.J., Hart J.S., Walker K.F. (1946). Lethal temperature relations for a sample of young speckled trout. Biol. Ser. Univ. Tor. Stud. Can..

[46] Reynolds W.W., Casterlin M.E. (1979). Behavioral thermoregulation and the “final preferendum” paradigm. Am. Zool..

[47] Fry F.E.J., Hoar W.S., Randall D.J. (1971). The effect of environmental factors on the physiology of fish. Fish Physiology.

[48] Elliot J.M. (1981). Some aspects of thermal stress in freshwater teleosts. Stress in Fish, Pickering, A.D., Ed..

[49] Jobling M. (1981). Temperature tolerance and the final preferendum—rapid methods for the assessment of optimum growth temperatures. J. Fish. Biol..

[50] Stevens D.L., Olsen A.R. (2004). Spatially balanced sampling of natural resources. J. Am. Stat. Assoc..

[51] Harris J.E., Silver G.S., Jolley J.C., Nelle R.D., Whitesel T.A. (2020). A stepwise approach to assess the occupancy state of larval lampreys in streams. J. Fish Wildl. Manag..

[52] Slade J.W., Adams J.V., Christie G.C., Cuddy D.W., Fodale M.F., Heinrich J.W., Quinlan H.R., Weise J.G., Weisser J.W., Young R.J. (2003). Techniques and methods for estimating abundance of larval and metamorphosed sea lampreys in Great Lakes tributaries, 1995 to 2001. J. Great Lakes Res..

[53] Eberhardt L.L. (1976). Quantitative ecology and impact assessment. J. Environ. Manag..

[54] Smith E.P. (2002). BACI design. Encycl. Environ..

[55] United States Bureau of Reclamation (BOR) Stream Gauge Data at Pendleton, Oregon (PDTO). https://www.usbr.gov/pn/hydromet/arcread.html.

[56] Sokal R.R., Rohlf F.J. (1995). Biometry.

[57] Smirnov A.K., Golovanov V.K., Zvezdin A.O., Golovanova I.L., Kucheryavyy A.V. (2020). Unusual thermoregulatory behavior of anadromous and resident larvae of the river lamprey *Lampetra fluviatilis* (Petromyzontidae). Inland. Water Biol..

[58] Selong J.H., McMahon T.E., Zale A.V., Barrows F.T. (2001). Effect of temperature on growth and survival of bull trout, with application of an improved method for determining thermal tolerance in fishes. Trans. Am. Fish. Soc..

[59] Munoz N.J., Anttila K., Chen Z., Heath J.W., Farrell A.P., Neff B.D. (2014). Indirect genetic effects underlie oxygen-limited thermal tolerance within a coastal population of chinook salmon. Proc. R. Soc. B Biol. Sci..

[60] Richter A., Kolmes S.A. (2005). Maximum temperature limits for Chinook, coho, and chum salmon, and steelhead trout in the Pacific Northwest. Rev. Fish. Sci..

[61] Mayer N.B., Hinch S.G., Eliason E.J. (2024). Thermal tolerance in Pacific salmon: A systematic review of species, populations, life stages and methodologies. Fish Fish..

[62] Spice E.K., Goodman D.H., Reid S.B., Docker M.F. (2012). Neither philopatric nor panmictic: Microsatellite and mtDNA evidence suggests lack of natal homing but limits to dispersal in Pacific lamprey. Mol. Ecol..

[63] Golovanov V.K., Nekrutov N.S., Zvezdin A.O., Smirnov A.K., Tsimbalov I.A. (2019). Thermoadaptation characteristics of European river lamprey *Lampetra fluviatilis* smolts. J. Ichthyol..

[64] United States Geological Survey (USGS) Real-Time Water Quality. https://waterwatch.usgs.gov/wqwatch/.

[65] Sloat M.R., Osterback A.M.K. (2013). Maximum stream temperature and the occurrence, abundance, and behavior of steelhead trout (*Oncorhynchus mykiss*) in a southern California stream. Can. J. Fish. Aquat. Sci..

[66] Speers-Roesch B., Norin T. (2016). Ecological significance of thermal tolerance and performance in fishes. Funct. Ecol..

[67] Roeder K.A., Roeder D.V., Bujan J. (2021). Ant thermal tolerance: A review of methods, hypotheses, and sources of variation. Ann. Entom. Soc. Am..

[68] Leong C.M., Tsang T.P., Guénard B. (2022). Testing the reliability and ecological implications of ramping rates in the measurement of Critical Thermal maximum. PLoS ONE.

[69] Sutphin Z.A., Hueth C.D. (2010). Swimming performance of larval Pacific lamprey (*Lampetra tridentata*). Northw. Sci..

[70] McCarthy M.A., Moore J.L., Morris W.K., Parris K.M., Garrard G.E., Vesk P.A., Rumpff L., Giljohann K.M., Camac J.S., Bau S.S. (2013). The influence of abundance on detectability. Oikos.

[71] MacKenzie D.I., Kendall W.L. (2002). How should detection probability be incorporated into estimates of relative abundance?. Ecol..

[72] MacKenzie D.I., Nichols J.D. (2004). Occupancy as a surrogate for abundance estimation. Anim. Biodiv. Conserv..

[73] Wenger S.J., Freeman M.C. (2008). Estimating species occurrence, abundance, and detection probability using zero-inflated distributions. Ecology.

[74] Holmes J.A., Lin P. (1994). Thermal niche of larval sea lamprey, Petromyzon marinus. Can. J. Fish. Aquat. Sci..

[75] Rodriguez-Munoz R., Nicieza A.G., Brana F. (2003). Density-dependent growth of sea lamprey larvae: Evidence for chemical interference. Funct. Ecol..

[76] Ebersole J.L., Liss W.J., Frissell C.A. (2001). Relationship between stream temperature, thermal refugia and rainbow trout *Oncorhynchus mykiss* abundance in arid-land streams in the northwestern United States. Ecol. Freshw. Fish.

[77] Hester E.T., Doyle M.W., Poole G.C. (2009). The influence of in-stream structures on summer water temperatures via induced hyporheic exchange. Limnol. Oceanog..

[78] Geist D.R., Hanrahan T.P., Arntzen E.V., McMichael G.A., Murray C.J., Chien Y.J. (2002). Physicochemical characteristics of the hyporheic zone affect redd site selection by chum salmon and fall Chinook salmon in the Columbia River. N. Am. J. Fish. Manag..

[79] Lutterschmidt W.I., Hutchison V.H. (1997). The critical thermal maximum: History and critique. Can. J. Zool..

[80] Beitinger T.L., Bennett W.A. (2000). Quantification of the role of acclimation temperature in temperature tolerance of fishes. Environ. Biol. Fish..

[81] Spigarelli S.A., Thommes M.M., Beitinger T.L. (1977). The influence of body weight on heating and cooling of selected Lake Michigan fishes. Comp. Biochem. Physiol. Part A Physiol..

[82] Muñoz N.J., Farrell A.P., Heath J.W., Neff B.D. (2015). Adaptive potential of a Pacific salmon challenged by climate change. Nat. Clim. Change.

[83] Anlauf-Dunn K., Kraskura K., Eliason E.J. (2022). Intraspecific variability in thermal tolerance: A case study with coastal cutthroat trout. Conser. Physiol..

[84] Sutherby J. (2019). The Effect of Temperature on Sea Lamprey (*Petromyzon marinus*): Ecological and Cellular Implications. Master’s Thesis.

